# Supplementary material for: Asynchronous magnetic resonance elastography: Shear wave speed reconstruction using noise correlation of incoherent waves
Source: Magn Reson Med. 2022 Oct 27;89(3):990–1001. doi: 10.1002/mrm.29502 (PMC9792433; doi:10.1002/mrm.29502)
Supplement: Supplementary file 1 — DATA S1 MATLAB code used to generate the numerical simulations of Figure 1 [file MRM-89-990-s002.zip › k-Wave/helpfiles/example_diff_homogeneous_medium_diffusion.html]

Heat Diffusion In A Homogeneous Medium (k-Wave)


# Heat Diffusion In A Homogeneous Medium

This example provides a simple demonstration of using k-Wave for the simulation of heat diffusion within a two-dimensional heterogeneous medium. It builds on the Homogenous Propagation Medium example.

- Open the file in the MATLAB Editor
- Run the file in MATLAB

## Contents

- Overview
- Defining the medium properties
- Defining the initial temperature distribution
- Running the simulation
- Comparison with exact solution

## Overview

In addition to the simulation functions for modelling acoustic and elastic waves, k-Wave also includes functions for the time domain simulation of heat diffusion and perfusion in heterogeneous media. The main simulation tool is called `kWaveDiffusion`, which is implemented as a MATLAB class. Similar to the other simulation functions in k-Wave, the constructor for `kWaveDiffusion` takes four input structures. These define the properties of the computational grid, the material properties of the medium, the properties and locations of any thermal sources, and the properties and locations of the sensor points used to record the evolution of the temperature field over time. The `kgrid` and `sensor` inputs are defined in the same way as the fluid and elastic wave codes, while the medium and source input structures have different field names related to the coefficients in the bioheat equation.

## Defining the medium properties

`kWaveDiffusion` solves Pennes' bioheat equation, so there are two sets of medium properties that can be defined. The first set is related to the diffusion of heat (conduction to other parts of the medium), and the second set is related to perfusion (the removal of heat by blood circulating in the tissue). In this example, only the diffusion properties are set. These can be defined in two ways, either by defining a single diffusion coefficient (assigned to `medium.diffusion_coeff`), or by defining the density, specific heat capacity, and thermal conductivity of the tissue (assigned to `medium.density`, `medium.specific_heat`, and `medium.thermal_conductivity`). All parameters can be specified as single scalar values in SI units (for homogeneous coefficients), or as matrices the same size as the computational grid (for heterogeneous coefficients). In this example, the individual medium properties are set as scalar values.

```
% define medium properties
medium.density              = 1079;     % [kg/m^3]
medium.thermal_conductivity = 0.52;     % [W/(m.K)]
medium.specific_heat        = 3540;     % [J/(kg.K)]
```

## Defining the initial temperature distribution

`kWaveDiffusion` allows two source terms to be defined. These are the initial temperature distribution within the medium, and the volume rate of heat deposition. The initial temperature is specified by assigning a single scalar value or a matrix (the same size as the computational grid) to `source.T0`. A heat source can also be specified in the same way by defining `source.Q` (the volume rate of heat deposition). In this example, the initial temperature distribution is set to be a Gaussian distribution positioned in the centre of the grid.

```
% set Gaussian initial temperature distribution [degC]
width = 4 * dx;
source.T0 = 37 + 5 .* exp( -(kgrid.x ./ width).^2 - (kgrid.y ./width).^2 );
```

## Running the simulation

The acoustic and elastic simulation functions in k-Wave (e.g., `kspaceFirstOrder2D` and `pstdElastic2D`) immediately execute when they are called. `kWaveDiffusion` is different in that it is defined as a class. Instead of running the simulation when the function is called, it returns an object of the `kWaveDiffusion` class, which can then be used to perform the simulation, or modify the settings (e.g., the properties of the source). The simulation is performed by calling the `takeTimeStep` method. By default, a visualisation of the temperature field is displayed during the simulation. The final temperature distribution can then be accessed using the `T` property of the object. Similar to the other simulation functions in k-Wave, optional inputs can also be used to modify the default behaviour.

```
% set input args
input_args = {'PlotScale', [37, 40]};

% create kWaveDiffusion object
kdiff = kWaveDiffusion(kgrid, medium, source, [], input_args{:});

% take time steps (temperature can be accessed as kdiff.T)
Nt = 300;
dt = 0.5;
kdiff.takeTimeStep(Nt, dt);

% plot the current temperature field
figure;
kdiff.plotTemp;
```

In this example, a `kWaveDiffusion` object is created without a sensor (this means only the current temperature is stored). The optional input `'PlotScale'` is used to show the temperature between 37 and 40 degrees. The simulation is then executed by defining the number and size of the time steps and calling `takeTimeStep`. The temperature after the simulation is complete is then displayed by calling the `plotTemp` method. The final temperature is displayed below.


## Comparison with exact solution

When the medium is homogeneous, the computation by `kWaveDiffusion` is exact and unconditionally stable. For a homogeneous medium, k-Wave also includes a second simulation function called `bioheatExact` which computes the exact solution to Pennes' bioheat equation at a given time in a single step. In this example, the final temperature distribution using the two codes agrees to machine precision.

```
% calculate diffusivity from medium parameters
D = medium.thermal_conductivity / (medium.density * medium.specific_heat);

% compute Green's function solution using bioheatExact
T_exact = bioheatExact(source.T0, 0, [D, 0, 0], kgrid.dx, Nt * dt);
```
